# Effects of rearing systems (cage versus floor) on the microbial composition and transcriptome of goose ileum

**DOI:** 10.3389/fvets.2024.1394290

**Published:** 2024-05-23

**Authors:** Zhiyu He, Xuejian Li, Xi Zhang, Qingyuan Ouyang, Jiwei Hu, Shenqiang Hu, Hua He, Liang Li, Hehe Liu, Jiwen Wang

**Affiliations:** ^1^Farm Animal Genetic Resources Exploration and Innovation Key Laboratory of Sichuan Province, Sichuan Agricultural University, Chengdu, China; ^2^State Key Laboratory of Swine and Poultry Breeding Industry, College of Animal Science and Technology, Sichuan Agricultural University, Chengdu, China

**Keywords:** goose, ileum, 16S rRNA sequencing, transcriptome, rearing system

## Abstract

There is a gradual transition from water to dryland rearing of geese. In this study, we performed 16S rRNA sequencing (16S rRNA-seq) and transcriptome sequencing (RNA-seq) to reveal the effects of cage rearing (CR) and floor rearing (FR) systems on the microbial composition and transcriptome of the goose ileum. Through 16S rRNA-seq, Linear Discriminant Analysis Effect Size (LEfSe) analysis identified 2 (*hgcI_clade* and *Faecalibacterium*) and 14 (*Bacteroides*, *Proteiniphilum*, *Proteiniclasticum*, etc.) differential microbiota in CR and FR, respectively. The rearing system influenced 4 pathways including biosynthesis of amino acids in ileal microbiota. Moreover, we identified 1,198 differentially expressed genes (DEGs) in the ileum mucosa, with 957 genes up-regulated in CR and 241 genes up-regulated in FR. In CR, Kyoto Encyclopedia of Genes and Genomes (KEGG) pathway analysis revealed the significant enrichment (*p* < 0.05) of 28 KEGG pathways, most of which were associated with amino acid metabolism. In FR, up-regulated DEGs were mainly enriched in KEGG pathways associated with cellular processes, including apoptosis, necroptosis, and cellular senescence. Spearman correlation analysis of differential microbiota and amino acid metabolism-related DEGs in CR showed a significant positive correlation. Additionally, differential microbiota of FR, *Phascolarctobacterium* and *Sutterella*, were positively correlated with *FGF10* (*p* < 0.05) and *PIK3R1* (*p* < 0.01), respectively. In conclusion, there might be differences in ileal amino acid metabolism levels between CR and FR geese, and the observed increase in harmful bacterial species in FR might impact the activity of ileal cells.

## 1 Introduction

Rearing systems constitute pivotal non-genetic factors that significantly impact productivity and individual health in goose farming (1). Cage rearing (CR) and floor rearing (FR) systems, as prevalent dryland rearing systems, can reduce the incidence of intestinal disease outbreaks caused by waterborne pathogens (2). However, knowledge on the effects of different dryland rearing systems on the intestines of geese is limited.

Most intestinal microbiota studies have focused on the cecum (3, 4) or the more accessible excreta (5, 6). However, the small intestine microbiota also plays an important role in host metabolic homeostasis (7, 8). The ileum, as a part of the small intestine, undertakes the final process of food digestion and absorption (9), and its microbial community is important for normal avian growth, including *Lactobacillus* and bacteria with butyrate-producing activity, like *Clostridium*, *Streptococcus* and *Enterococcus* (10). Moreover, compared to other intestinal segments, there is an abundance of immune cells in the ileum (11). Thus, the ileal microbiota may acts in the maintenance of intestinal health by constituting a well-developed immune system. Numerous studies have demonstrated that rearing systems exert significant influence on ileal development and microbial composition. In chickens, the rearing system was shown to alter the relative weight, microbial composition, and expression levels of immune factors (IL-1β, TNF-α, and IFN-γ) in the ileum (12). Ground litter broilers exhibited higher ileal microbiota α diversity (13), meanwhile, ground rearing increased the abundance of litter breeding bacteria (*Facklamia*, *Globicatella*, and *Jeotgalicoccus*) and potentially pathogenic bacteria (*Streptococcus* and *Staphylococcus*) in the ileum (14). In ducks, diverse floor rearing environments altered the dominant bacterial phyla in the ileum of Shaoxing ducks, with ducks reared on plastic mesh floor showing significantly higher ileal villus height and villus height/crypt depth ratio compared to those reared on litter floor (15). Importantly, dryland rearing on netting floors has been noted to enhance the intestinal immunity and reduce the mortality rate (2). Studies on Nonghua ducks demonstrated that floor rearing individuals had significantly higher relative weight, relative length and relative weight/relative length ratios of the ileum compared to net rearing individuals (16). In geese, previous research has indicated that cage rearing geese exhibited a higher villus height/crypt depth ratio, suggesting the potential benefits of cage rearing in enhancing geese resistance against diseases and toxins (17). The impact of rearing systems on avian intestinal microbial composition has been extensively studied, while there is a paucity of research focused on elucidating the effects of alterations in avian intestinal microbial composition induced by rearing systems on the intestinal transcriptome.

In this study, we compared the ileal microbial composition and ileal mucosal transcriptome of cage rearing (CR) and floor rearing (FR) geese to deepen our understanding of how rearing systems affect intestines, which provides a theoretical basis for the management of intestinal health in geese.

## 2 Materials and methods

### 2.1 Experiment animals and sample collection

All animal handling procedures were approved by the Institutional Animal Care and Use Committee (IACUC) of Sichuan Agricultural University (Chengdu campus, Sichuan, China, Permit No. DKY20170913).

The same batch of male goslings, from the Sichuan Agricultural University Waterfowls Breeding Farm (Ya’an, Sichuan, China), were reared under the same rearing environment with free access to feed and water until 120 days. Afterwards, they were randomly divided into 2 groups: CR and FR. At 270 days old, 8 geese were randomly selected for slaughter from each group. The experimental geese were euthanised by carbon dioxide inhalation and cervical dislocation after approximately 12 h of fasting. The intestinal digesta and mid-ileum mucosa were quickly collected and rapidly frozen in liquid nitrogen. Digesta was used for 16S rRNA-seq and mucosa was used for RNA-seq, and both were stored at −80°C prior to sequencing.

### 2.2 DNA extraction and 16S rRNA sequencing

In this study, microbial DNA extraction was conducted using the E.Z.N.A. Stool DNA Kit (Omega Bio-Tek, Norcross, GA). DNA concentration and purity were characterized by NanoDrop spectrophotometer (Thermo Fisher Scientific, Waltham, MA, United States) and electrophoresis on 1% agarose gels. DNA with (OD260/OD280) range from 1.8 to 2.0 and (OD260/OD230) range from 2.0 to 2.5 could be used for subsequent experiments. Based on the concentration, DNA was diluted to 1ug/μL with sterile water. And, the V3−V4 hypervariable region was targeted for amplification, utilizing primers 338-F (5′-ACTCCTACGGGAGGCAGCAG-3′) and 806-R (5′-GGACTACNNGGGTATCTAAT-3′). The PCR reaction was performed on a thermocycling PCR system (Bio-Rad T100, Germany) using high-fidelity polymerase according to the following procedure: 98°C for 60 s; 30 cycles of 98°C for 10 s, 50°C for 30 s, and 72°C for 30 s; 72°C for 5 min. The same volume of IX loading buffer (contained SYB green) was mixed with the PCR products and detected by electrophoresis on 2% agarose gels. The PCR products were mixed at an equidensity ratio. Then, the mixture PCR products were purified with Qiagen Gel Extraction Kit (Qiagen, Germany). Following the manufacturer’s recommendations, sequencing libraries were generated using TruSeq^®^ DNA PCR-Free Sample Preparation Kit (Illumina, United States) and index codes were added. Library quality was assessed by Qubit@2.0 Fluorometer (Thermo Scientific) and Agilent Bioanalyzer 2100 system (Agilent Technologies, CA, United States). Purified amplicons were sequenced on Illumina NovaSeq 6000 platform (2 × 250 paired ends) by Novogene Co., Ltd. (Beijing, China).

### 2.3 Microbial bioinformatics analysis of the ileum

The assembly of paired-end reads, characterized by overlaps exceeding 10 bp, was accomplished using FLASH software (version 1.2.11) (18). Subsequently, reads of low quality, containing ambiguous characters and sequences shorter than 400 bp, were excluded. QIIME2 software (version 2023.5) (19) was employed for processing and assignment of these assembly readings. The denoise-paired method in DADA2 was applied to identify amplicon sequence variants (ASVs). Annotation of results utilized the SILVA 138 database (20), providing classifications at the kingdom, phylum, class, order, family and genus. QIIME2 (version 2023.5) facilitated the calculation of α and β diversity, and unweighted UniFrac distance metrics were used to generate principal coordinate analysis (PCoA). Differences in microbial composition between the 2 groups were assessed using Linear Discriminant Analysis Effect Size (LEfSe) (21), employing screening criteria of LDA score > 3 and *p* < 0.05. Additionally, we then predicted ileal microbial metabolic pathways using the PICRUSt2 (22) and used STAMP software (version 2.1.3) (23) to compare differences in function. Welch *t*-test (two-sided) was used for intergroup comparison, and the Welch’s inverted confidence interval (CI) method was used for CI calculation. *p* < 0.05 indicated a significant difference.

### 2.4 RNA isolation and sequencing

Total RNA extraction from the ileum was accomplished using Trizol (Invitrogen, Carlsbad, CA, United States), following the manufacturer’s instructions. RNA integrity was assessed using the Fragment Analyzer 5400 (Agilent Technologies, CA, United States). RNA integrity values range from 6.8 to 8.9. The library construction utilizing the obtained RNA was undertaken by Novogene Co., Ltd. (Beijing, China). All Illumina PE libraries were constructed and 2 × 150 bp RNA-seq was completed using the Illumina sequencing platform (NovaSeq 6000). The datasets presented in this study can be found in the National Center for Biotechnology Information (NCBI) under BioProject ID PRJNA1054312.

### 2.5 Transcriptome alignment and assembly

Standard quality control measures were implemented using Fastp software (version 0.23.1) (24) to filter out low-quality reads, ensuring the retention of clean reads for subsequent analyses. The obtained clean reads were aligned to the goose reference genome (BioProject ID PRJNA801885, data not released) using HISAT2 software (version 2.2.1) (25). The resulting SAM (sequencing alignment/mapping) files were subsequently converted to BAM (binary alignment/mapping) files and sorted using SAMtools (version 1.15.1) (26). The expression levels of each transcript were computed using featureCounts (version 2.0.3) (27), while gene expression was quantified using the transcripts per million (TPM) method.

### 2.6 Identification and functional analysis of differentially expressed genes

DEseq2 (28) was employed to identify differentially expressed genes (DEGs) between groups. Genes exhibiting |Log_2_(FC)| ≥ 1 and *p* < 0.01 were designated as DEGs. And the online tool KOBAS (version 3.0) (29) was utilized for Gene Ontology (GO) and Kyoto Encyclopedia of Genes and Genomes (KEGG) functional analysis, accessible at http://kobas.cbi.pku.edu.cn/kobas3/?t=1. The functional gene analysis was conducted using *G. gallus* as a reference.

### 2.7 Statistical analysis

Statistical analysis was performed using SPSS 27.0 software. Spearman’s correlation coefficients were calculated to analyze the correlation. Differences were considered statistically significant at *p* < 0.05.

## 3 Results

### 3.1 16S rRNA sequencing basic information and classification of amplicon sequence variants

**Figure 1:**
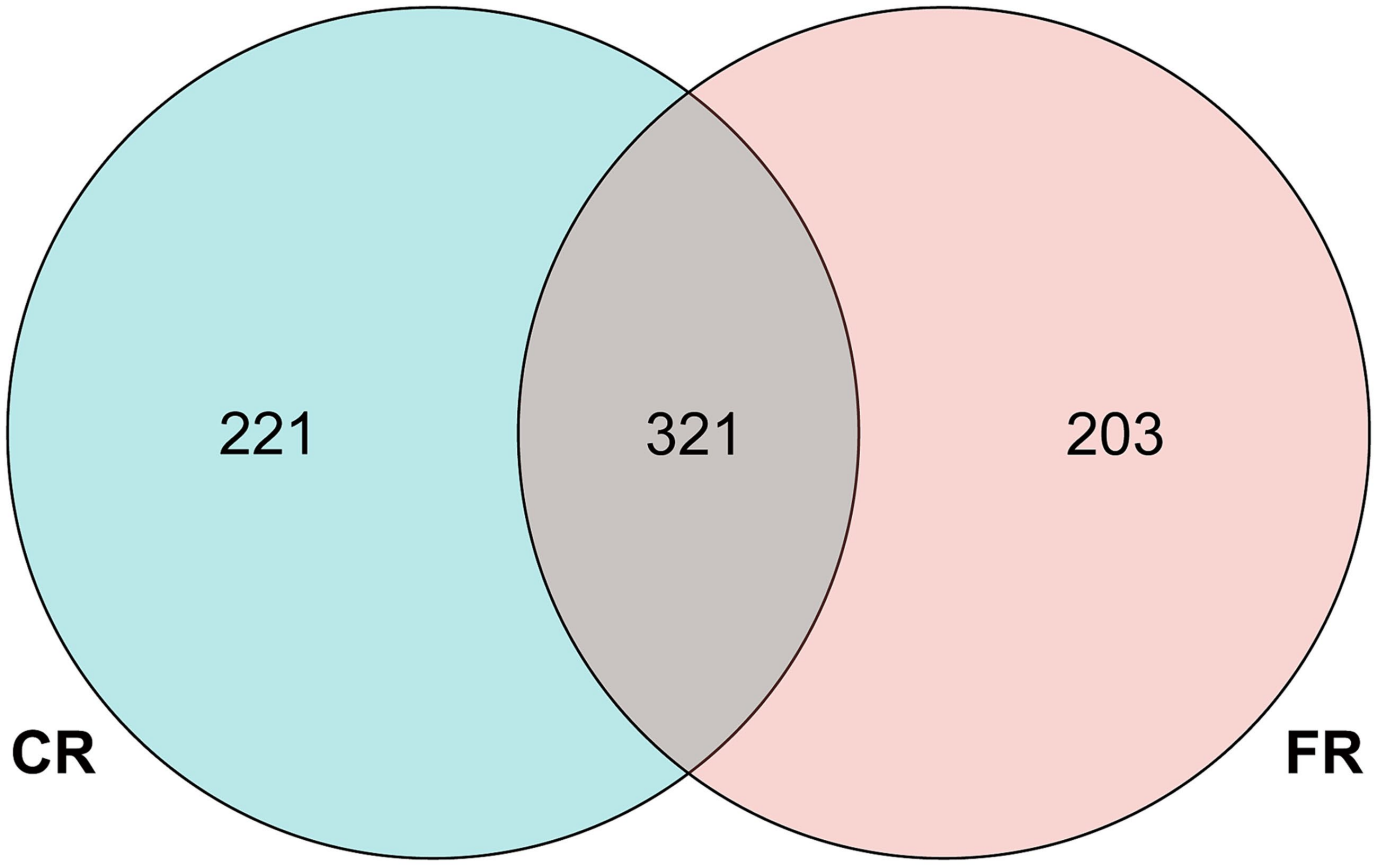
Venn diagram of the number of ASVs.

Following quality control and filtering procedures, a total of 957,019 effective reads were generated from 16 samples, averaging 59,814 reads per sample (Supplementary Table S1). A total of 745 ASVs were identified through QIIME2 analysis. Of these, 321 ASVs were found to be common to both rearing systems, while 221 ASVs were exclusive to the CR and 203 ASVs were unique to the FR (Figure 1). Subsequent taxonomic classification categorized these ASVs into 17 phyla, 34 classes, 88 orders, 167 families, 215 genera.

### 3.2 Ileal microbial composition and differential microbiota identification

**Figure 2:**
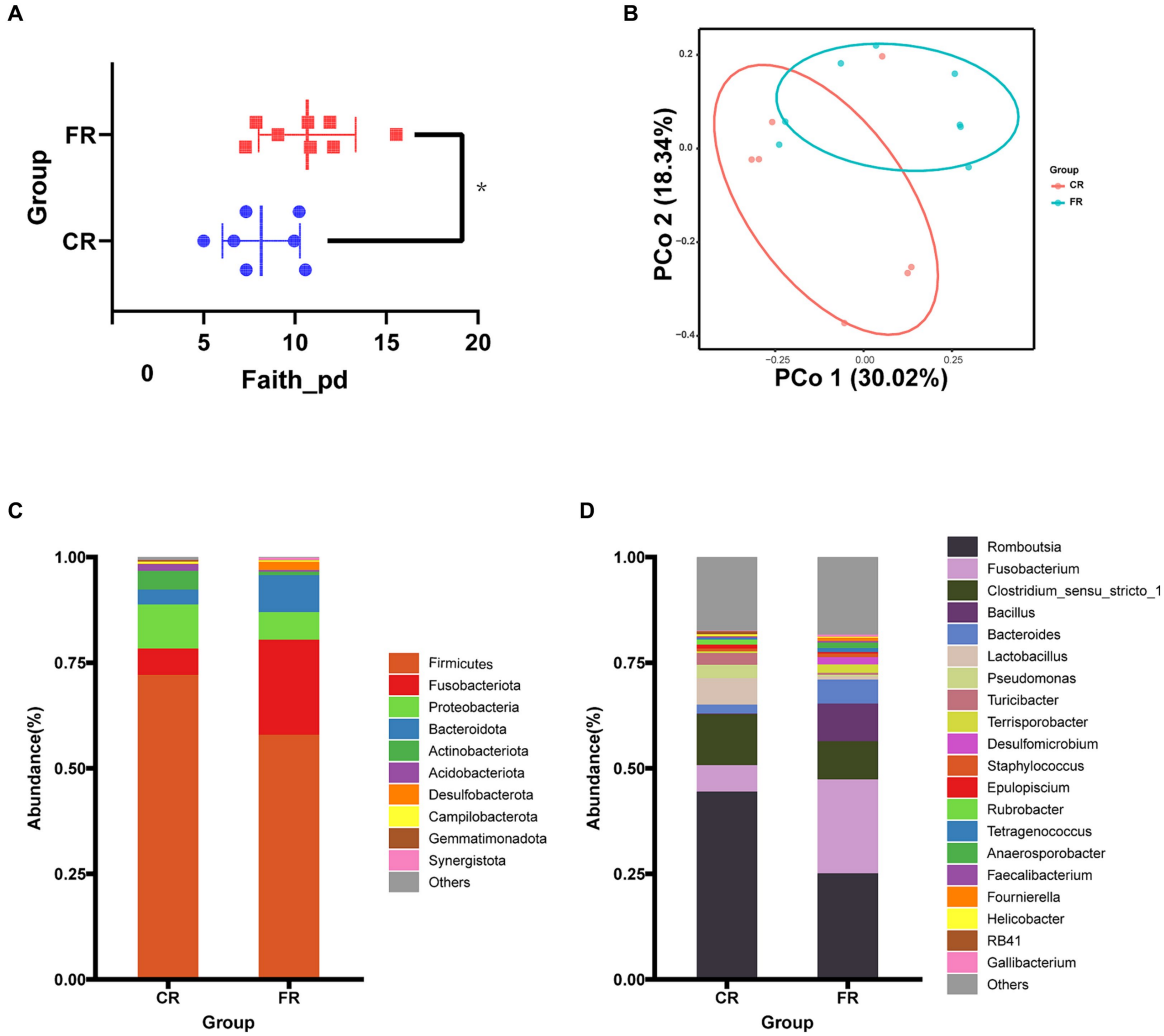
Ileal microbiological composition comparison between CR and FR. **(A)** Faith’s PD α-diversity. **(B)** The PCoA based on the unweighted UniFrac distance. **(C)** Phylum-level microbial composition. **(D)** Genus-level microbial composition.

The complexity of the ileal microbiota was estimated on the basis of α-diversity indices (Observed_features, Shannon, Chao1, Faith’s PD, Evenness). The analysis revealed that the Faith’s PD α-diversity of CR was significantly lower than that of FR (Figure 2A). PCoA analyses demonstrated that the first principal coordinate (PCo1) explained 30.02% of the variations among samples and the second principal coordinate (PCo2) explained 18.34% of the variations (Figure 2B). The ANOSIM test further confirmed significant differences in ileal microbial communities between CR and FR (*R* = 0.22, *p* = 0.04). Comparison analysis of ileal microbiota at the phylum level and genus levels revealed broad similarities in dominant microbiota between the 2 rearing systems. At the phylum level, the dominant phyla in CR were *Firmicutes* (72.11%), *Proteobacteria* (10.39%) and *Fusobacteriota* (6.27%); in FR the dominant phyla were *Firmicutes* (54.64%), *Fusobacteriota* (19.76%) and *Bacteroidetes* (10.62%) (Figure 2C). At the genus level, in CR the top 3 genera were *Romboutsia* (45.03%), *Clostridium_sensu_stricto_1* (12.26%) and *Fusobacterium* (6.78%); in FR, the top 3 genera were also *Romboutsia* (23.38%), *Fusobacterium* (21.13%) and *Clostridium_sensu_stricto_1* (8.85%) (Figure 2D).

**Figure 3:**
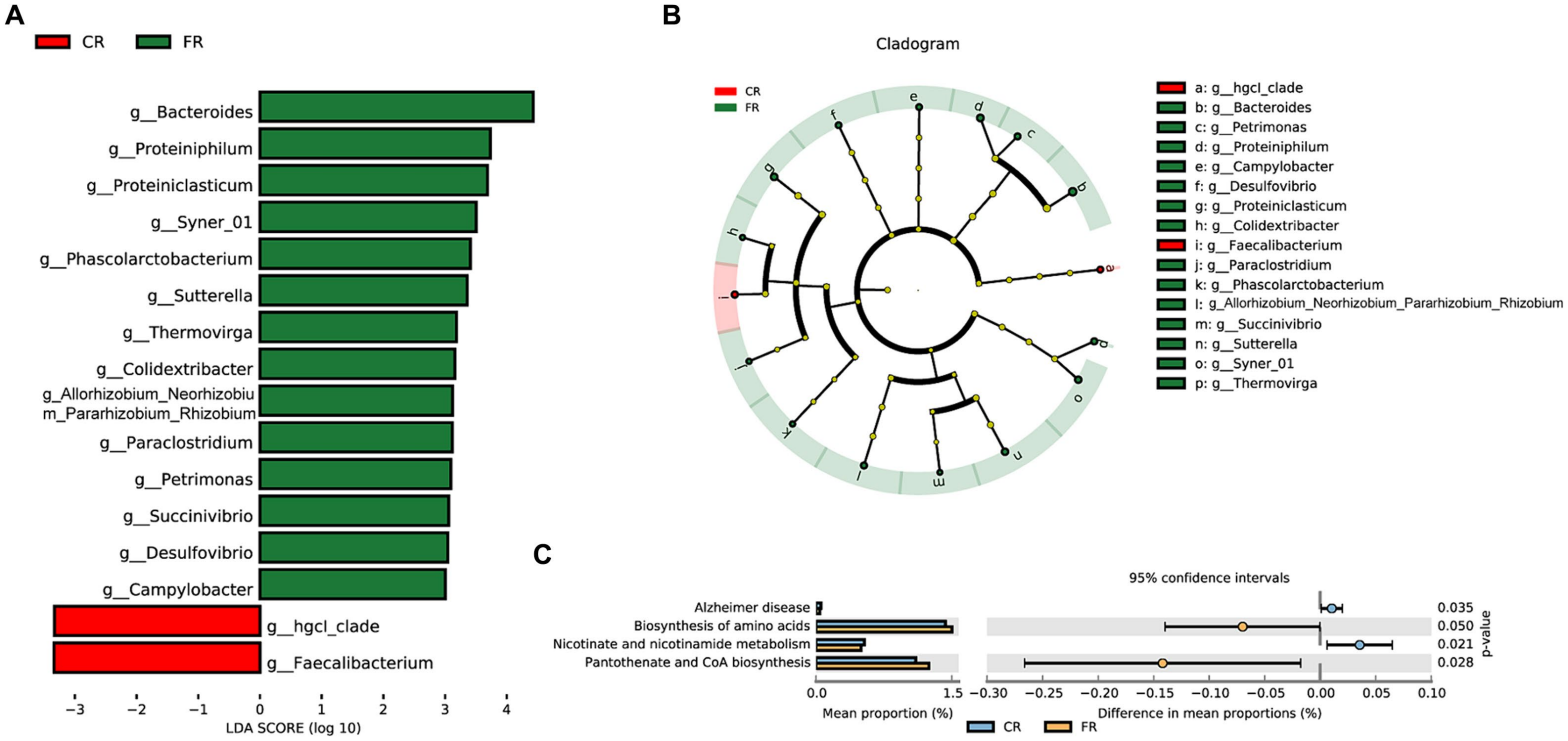
Differential microbiota identification and function prediction. **(A)** Differential microbiota in the CR and FR ileum. **(B)** Cladogram of differential microbiota. **(C)** Differential metabolic pathways predicted.

At the genus level, LEfSe analyses identified 2 and 14 differential microbiota from CR and FR, respectively (Figures 3A,B). In CR, the abundance of *hgcI_clade* and *Faecalibacterium* was significantly higher than that of FR. Meanwhile, *Bacteroides, Proteiniphilum, Proteiniclasticum, Syner_01, Phascolarctobacterium, Sutterella, Thermovirga, Colidextribacter, Allorhizobium_Neorhizobium_Pararhizobium_Rhizobium, Paraclostridium, Petrimonas, Succinivibrio, Desulfovibrio,* and *Campylobacter* had higher abundance in FR. In addition, PICRUSt2 predictive function analyses indicated that 4 metabolic pathways differed between the 2 groups, including Alzheimer disease, biosynthesis of amino acids, nicotinate and nicotinamide metabolism and pantothenate and CoA biosynthesis (Figure 3C).

### 3.3 Overview of transcriptome sequencing and identification of the differentially expressed genes

**Figure 4:**
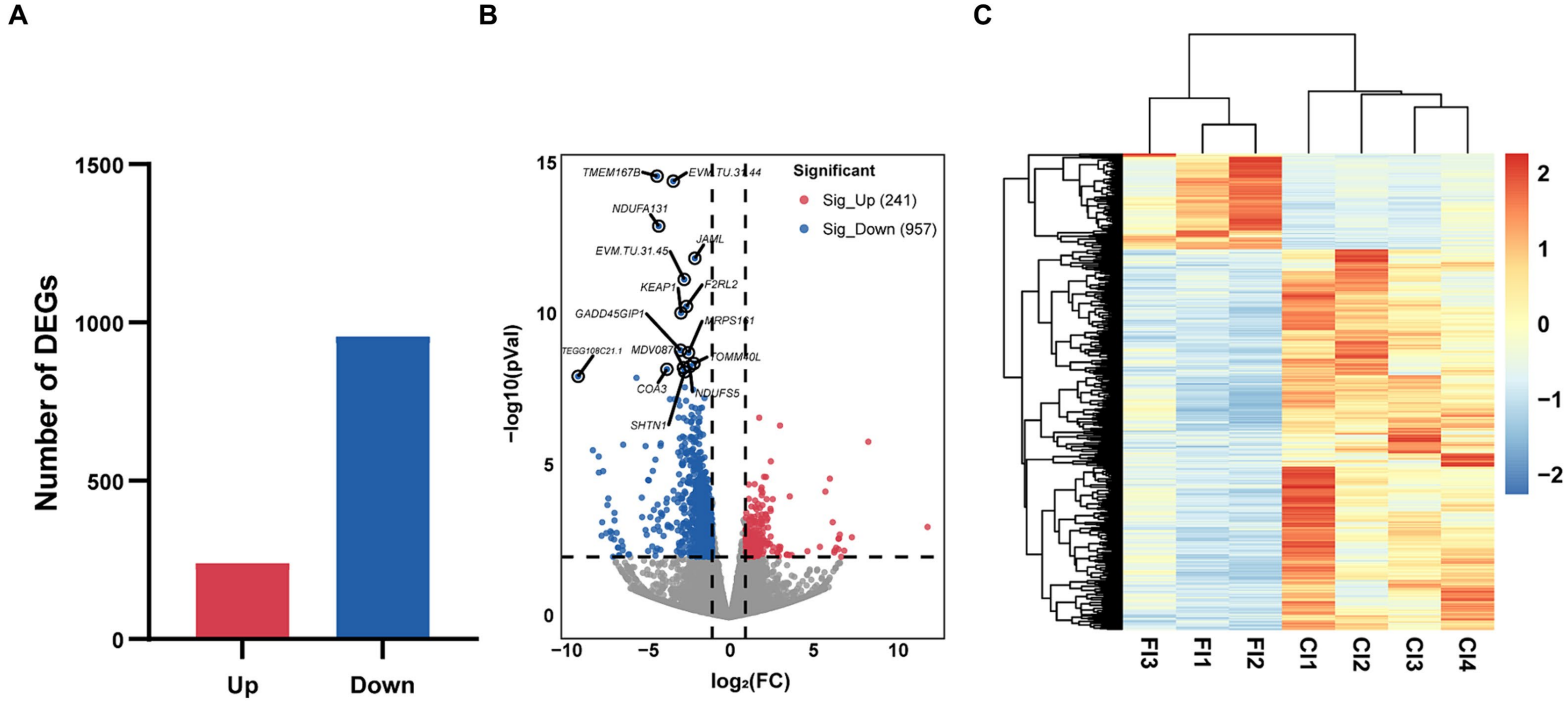
Characterizations of ileal transcriptome variation between CR and FR. **(A)** The number of DEGs. **(B)** Volcano map. The red dots represent up-regulated genes and blue dots represent down-regulated genes. **(C)** Hierarchical clustering of DEGs. DEG, differentially expressed gene; CI, ileum of cage rearing geese; FI, ileum of floor rearing geese.

A comprehensive set of 292,451,214 raw reads were generated across the 7 samples, and subsequent stringent filtering yielded an average of 40,961,820 clean reads for each sample. The quality metrics, including Q20, Q30, GC content, and mapping rates, exhibited favorable ranges of 97.15–97.81%, 92.82–94.08%, 44.72–52.03%, and 86.86–93.52%, respectively (Supplementary Table S2). These results attested to the robust sequencing quality essential for subsequent analyses. We identified a total of 1,198 DEGs (Figure 4A), of which 241 were up-regulated and 957 were down-regulated (Figure 4B). The clustering heatmap of TPM illustrated the expression profiles of DEGs in the ileum of CR and FR geese in a visually informative manner (Figure 4C).

### 3.4 Functional enrichment analysis of differentially expressed genes

**Figure 5:**
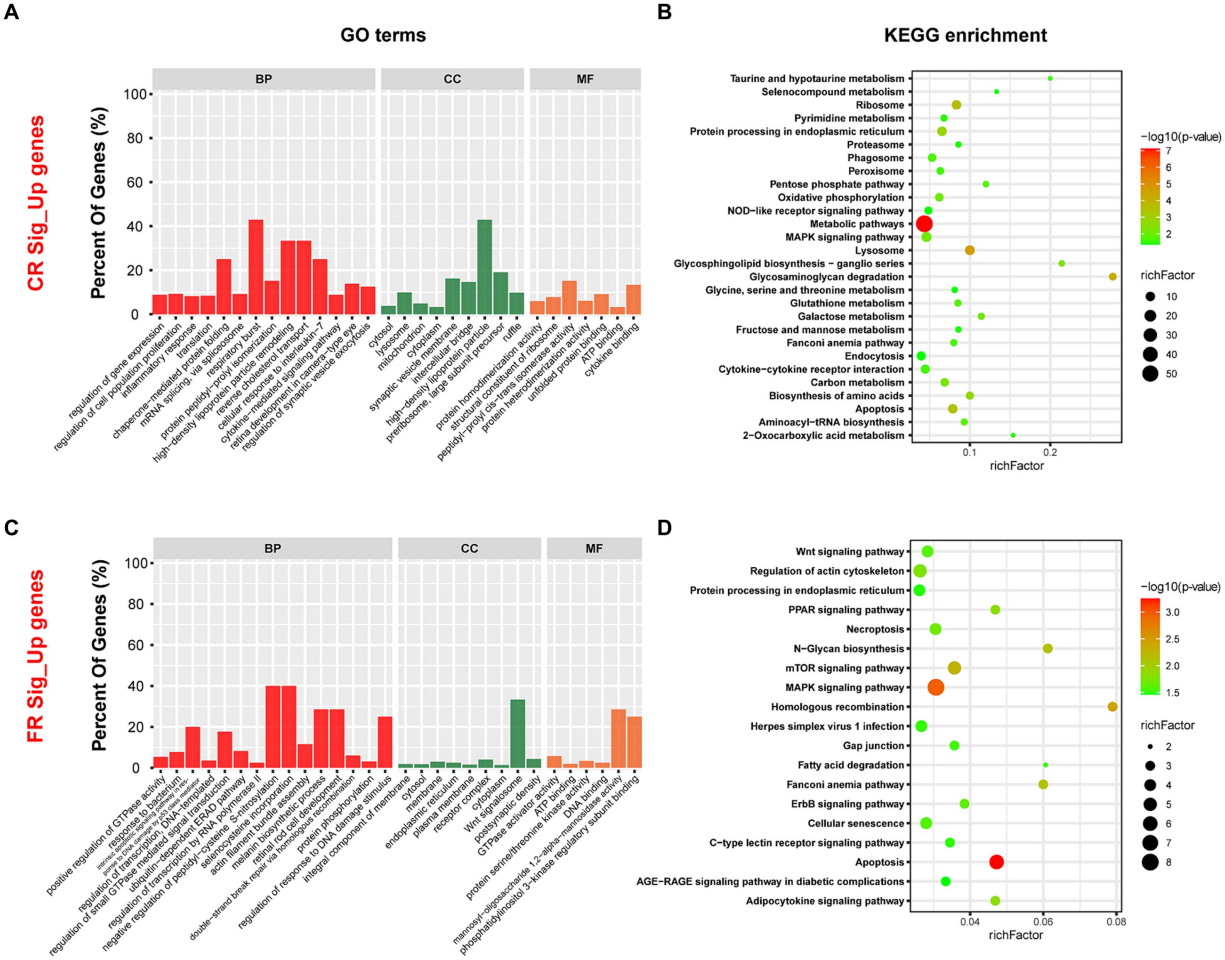
GO terms and KEGG pathways enriched by DEGs. **(A)** Top 30 GO terms enriched by DEGs up-regulated in CR. **(B)** The KEGG pathways significantly enriched by DEGs up-regulated in CR. **(C)** Top 30 GO terms enriched by DEGs up-regulated in FR. **(D)** The KEGG pathways significantly enriched by DEGs up-regulated in FR. GO, Gene Ontology; KEGG, Kyoto Encyclopedia of Genes and Genomes; BP, biological processes; CC, cellular components; MF, molecular functions.

Compared to FR, 957 genes were up-regulated in CR. These genes were enriched to 220 GO terms, including 129 biological processes (BP), 44 cellular components (CC), and 47 molecular functions (MF) (*p* < 0.05) (Figure 5A). The top 3 GO terms were cytosol, protein homodimerization activity, and lysosome. Based on KEGG pathway enrichment analysis, 28 pathways were identified significantly (*p* < 0.05) (Figure 5B). Metabolic pathways, lysosome, and glycosaminoglycan degradation were included. Further analysis revealed that 15 of the 28 pathways were related to metabolism, 5 to genetic information processing, 5 to cellular processes, 2 to environmental information processing, and 1 to organismal systems. Metabolism was the most up-regulated function, with 46.67% of the pathways associated with amino acid metabolism. These pathways included glycosaminoglycan degradation, biosynthesis of amino acids, glycosphingolipid biosynthesis—ganglio series, glutathione metabolism, taurine and hypotaurine metabolism, selenocompound metabolism, and glycine, serine and threonine metabolism.

Compared to CR, 241 genes were up-regulated in the FR and they included 119 BP, 39 CC, and 41 MF (*p* < 0.05) (Figure 5C). Integral component of membrane, cytosol, and GTPase activator activity were the GO terms with the highest significance. Through KEGG enrichment analysis, we identified 19 signaling pathways (*p* < 0.05), with the highest percentage of pathways associated with cellular processes (Figure 5D). Pathways associated with cellular processes included apoptosis, regulation of actin cytoskeleton, necroptosis, cellular senescence, and gap junction were up-regulated in FR.

### 3.5 Correlation analysis of differential microbiota and key differentially expressed genes

**Figure 6:**
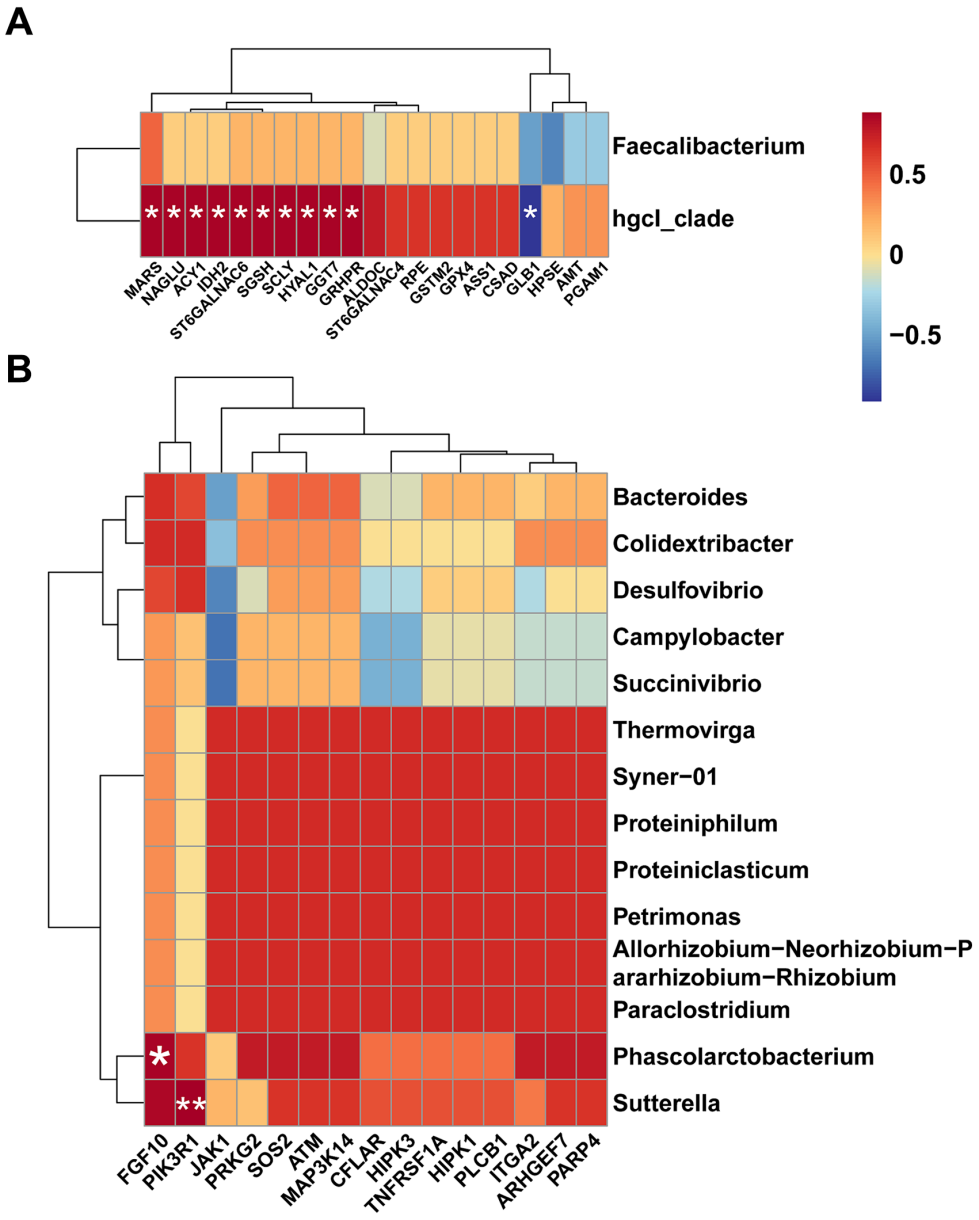
Heat maps for Spearman correlation analysis. **(A)** Spearman correlation analysis of differential microbiota and amino acid metabolism-related DEGs in CR. **(B)** Spearman correlation analysis of differential microbiota and cellular processes-related DEGs in FR.

Combining these findings, we generated heat maps based on the results of Spearman correlation analysis. Illustrated in Figure 6A, we performed Spearman correlation analysis between up-regulated DEGs involved in amino acid metabolism and differential microbiota in CR. The abundance of *hgcI_clade* was significant positively correlated with the expression of *MARS, NAGLU, ACY1, IDH2, ST6GALNAC6, HYAL1, SGSH, SCLY, GGT7*, and *GRHPR*, while demonstrating a significant negative correlation with the expression of *GLB1* (*p* < 0.05). Simultaneously, we analyzed the correlation between up-regulated DEGs involved in cellular processes and differential microbiota in FR, and the results showed that *FGF10* was significantly and positively correlated with *Phascolarctobacterium* (*p* < 0.05), and, *PIK3R1* was significantly and positively correlated with *Sutterella* (*p* < 0.01) (Figure 6B).

## 4 Discussion

The intestinal microbiota of animals represents a complex and dynamic entity susceptible to environmental influences (8). Several studies have highlighted the profound impact of rearing systems on avian intestinal development (30) and microbial composition (4, 31, 32), thereby influencing intestinal functions, digestion, and nutrient absorption (33). However, the consequences of altered microbial composition on intestinal transcriptome have received limited attention.

Consistent with previous reports (34), the rearing system was found to impact ileal microbial composition. Comparison of the microbial composition in CR and FR revealed dominant phyla such as *Firmicutes*, *Proteobacteria*, *Fusobacteriota*, and *Bacteroidetes*, consistent with previous goose studies (35). *Firmicutes*, *Bacteroidetes*, and *Proteobacteria* have also been identified as the major phyla in the intestinal tracts of chickens and turkeys, collectively constituting over 90% of all sequences (36). Additionally, *Romboutsia* emerged as the primary genus in both rearing systems, a finding somewhat divergent from certain investigations into ileal microbial composition in chickens (37, 38). Notably, *Romboutsia* has been identified as the predominant genus in the ileum of cage rearing chickens, potentially linked to body weight maintenance (39), lipid metabolism (40), and intestinal water metabolism (41). Further analysis revealed 2 and 14 differential microbiota in CR and FR, respectively.

Differential microbiota identified in CR, *hgcI_clade*, belongs to *Actinobacteria* (42), which has been repeatedly reported to be present in water bodies (43, 44). Some reports considered it as a potential probiotic (45). The results of Spearman’s correlation analysis showed, *hgcI_clade* was significant correlated with *MARS, NAGLU, ACY1, IDH2, ST6GALNAC6, HYAL1, SGSH, SCLY, GGT7, GRHPR*, and *GLB1*. This suggested that *hgcI_clade* may influence amino acid metabolism by modulating enzyme levels. *MARS*, encoding the methionyl-tRNA synthetase, may play a role in regulating the cell cycle by linking methionine and cyclin-dependent kinase 4 (46). The *NAGLU* encodes an enzyme involved in the catabolism of glycosaminoglycans through hydrolysis of the terminal N-acetyl-D-glucosamine residue in N-acetyl-alpha-D-glucosaminides (47). A Study on rats demonstrated that the expression of *ACY1* (aminoacylase 1) was associated with the development of the jejunal crypt-villus axis and could be used as a marker for the metabolism of intestinal N-α-acetylated protein metabolism (48). Isocitrate dehydrogenase-2 (*IDH2*) is a marker of mitochondrial function (49), and its main function is catalyzing the oxidative decarboxylation of isocitrate to producing α-ketoglutarate (50). The *ST6GALNAC6* gene encodes ST6 N-acetylgalactosaminide α-2,6-sialyltransferase 6, an enzyme belonging to the family of sialyltransferase that may catalyze the addition of sialic acid to N-acetylgalactosamine via an α-2,6 linkage (51), and its expression is associated with colon health (52). Degradation of intracellular hyaluronan acid is the main function of hyaluronidase 1 (*HYAL1*), which affects cell proliferation, migration and differentiation (53) and participates in neuroimmunomodulators in the microbiota-gut axis (54). *SGSH, SCLY, GGT7, GRHPR*, and *GLB1*, encoding N-sulfoglucosamine sulfohydrolase (55), selenocysteine lyase (56), gamma-glutamyltransferase 7 (57), glyoxylate and hydroxypyruvate reductase (58), and galactosidase beta 1 (59), are involved in various processes of amino acid metabolism. Another differential microbiota in CR, *Faecalibacterium* is known to exert vital effects in immune system regulation, intestinal barrier protection, and microbiota regulation (60). Recent findings have shown that spermidine produced by *Faecalibacterium* could improve intestinal function in geese (61) and chickens (62). Integrating the insights from our previous studies (17) and the current study, *Faecalibacterium* may be involved in enhancing ileal development in CR geese.

In our study, the FR system appeared to increase the number of harmful genera in the goose ileum, such as *Campylobacter*, *Sutterella*, *Paraclostridium*, *Succinivibrio*, and *Desulfovibrio*. *Campylobacter* is a common cause of gastroenteritis in humans worldwide (63) and poultry is the primary host (64, 65). Our findings suggested that cage rearing is essential for reducing *Campylobacter* colonization in the ileum, thus preventing contamination of goose products (66). *Sutterella*, a gram-negative microaerophilic bacterium, has been implicated in various human diseases such as autism (67, 68), Down syndrome (69) and inflammatory bowel disease (70). And it has been found in the liver and breast of chickens, which could be a potential source of contamination for humans (71). Some species of *Paraclostridium* have been linked to fatal infections in humans and animals (72), but the mechanism is unknown (73). It was certain that *Paraclostridium* was associated with poultry meat spoilage and was difficult to eradicate (74). *Succinivibrio*, belonging to the family *Succinivibrionaceae*, ferments glucose and other carbohydrates to produce large amounts of acetic acid and succinic acid (75), which might have pro-inflammatory effects (76). Studies in pigs have implicated *Desulfovibrio* as a major contributor in the utilization of feces for H_2_S production (77). And, H_2_S has been hypothesized to contribute to intestinal diseases such as inflammatory bowel disease (78), particularly ulcerative colitis (79). The RNA-seq results seemed to reflect the effect of harmful genera on ileal function. Because, the increase of these bacteria up-regulated pathways associated with apoptosis, necroptosis, and cellular senescence, suggesting adverse effects on ileal cells (80, 81).

## 5 Conclusion

In conclusion, there might be differences in ileal amino acid metabolism levels between CR and FR geese. Besides, the increase in harmful bacterial species in FR might affect the activity of ileal cells. This study provides new insights into the selection of appropriate dryland rearing systems to maintain intestinal health in geese. And more comprehensive molecular regulatory networks remain to be further investigated.

## Data availability statement

The data presented in the study are deposited in the National Center for Biotechnology Information (NCBI) repository, accession number PRJNA1054312.

## Ethics statement

The animal study was approved by the Institutional Animal Care and Use Committee (IACUC) of Sichuan Agricultural University (Chengdu campus, Sichuan, China, Permit No. DKY20170913). The study was conducted in accordance with the local legislation and institutional requirements.

## Author contributions

ZH: Data curation, Formal analysis, Investigation, Methodology, Writing – original draft. XL: Conceptualization, Funding acquisition, Methodology, Project administration, Resources, Supervision, Writing – review & editing. XZ: Data curation, Investigation, Methodology, Writing – review & editing. QO: Data curation, Investigation, Methodology, Writing – review & editing. JH: Data curation, Investigation, Methodology, Writing – review & editing. SH: Resources, Writing – review & editing. HH: Data curation, Investigation, Methodology, Writing – review & editing. LL: Investigation, Writing – review & editing. HL: Investigation, Writing – review & editing. JW: Funding acquisition, Writing – review & editing.
